# Analgesic efficacy of cerebral and peripheral electrical stimulation in chronic nonspecific low back pain: a randomized, double-blind, factorial clinical trial

**DOI:** 10.1186/s12891-015-0461-1

**Published:** 2015-01-31

**Authors:** Fuad Ahmad Hazime, Diego Galace de Freitas, Renan Lima Monteiro, Rafaela Lasso Maretto, Nilza Aparecida de Almeida Carvalho, Renata Hydee Hasue, Silvia Maria Amado João

**Affiliations:** Department of Physical Therapy, Universidade Federal do Piauí – UFPI, Avenida São Sebastião, 2819, CEP: 64202-020 Parnaíba, PI Brasil; Department of Physical Therapy, Irmandade Santa Casa de Misericórdia de São Paulo, Rua Dr Cesário Motta Jr, 112, CEP: 01221-020 São Paulo, SP Brasil; Department of Physical Therapy, Universidade Paulista – UNIP, Rua Antonio de Macedo, 505, CEP: 03087-040 São Paulo, SP Brasil; Department of Physical Therapy, Universidade Federal do Amapá – UFAP, Macapá, AP – Brasil, Rod. Juscelino Kubitschek Km 02, CEP: 68903-419 Macapá, AP Brasil; Department of Physical Therapy, Speech-Language Pathology and Occupational Therapy, Doctoral Programs in Rehabilitation Sciences, Faculdade de Medicina da Universidade de São Paulo – USP, Rua Cipotânea, 51, Cidade Universitária, CEP: 05360-160 São Paulo, SP Brasil

**Keywords:** Chronic back pain, Transcranial direct current stimulation, Transcutaneous electrical nerve stimulation, Randomized clinical trial, Factorial design

## Abstract

**Background:**

Chronic non-specific low back pain is a major socioeconomic public health issue worldwide and, despite the volume of research in the area, it is still a difficult-to-treat condition. The conservative analgesic therapy usually comprises a variety of pharmacological and non-pharmacological strategies, such as transcutaneous electrical nerve stimulation. The neuromatrix pain model and the new findings on the process of chronicity of pain point to a higher effectiveness of treatments that address central rather than peripheral structures. The transcranial direct current stimulation is a noninvasive technique of neuromodulation that has made recent advances in the treatment of chronic pain. The simultaneous combination of these two electrostimulation techniques (cerebral and peripheral) can provide an analgesic effect superior to isolated interventions. However, all the evidence on the analgesic efficacy of these techniques, alone or combined, is still fragmented. This is a protocol for a randomized clinical trial to investigate whether cerebral electrical stimulation combined with peripheral electrical stimulation is more effective in relieving pain than the isolated application of electrical stimulations in patients with chronic nonspecific low back pain.

**Methods/Design:**

Ninety-two patients will be randomized into four groups to receive transcranial direct current stimulation (real/sham) + transcutaneous electrical nerve stimulation (real/sham) for 12 sessions over a period of four weeks. The primary clinical outcome (pain intensity) and the secondary ones (sensory and affective aspects of pain, physical functioning and global perceived effect) will be recorded before treatment, after four weeks, in Month 3 and in Month 6 after randomization. Confounding factors such as anxiety and depression, the patient’s satisfaction with treatment and adverse effects will also be listed. Data will be collected by an examiner unaware of (blind to) the treatment allocation.

**Discussion:**

The results of this study may assist in clinical decision-making about the combined use of cerebral and peripheral electrical stimulation for pain relief in patients with chronic low back pain.

**Trial registration:**

NCT01896453

## Background

Low back pain is one of the main musculoskeletal complaints in clinics and hospitals and is still one of the six most often found health conditions in developed countries [1]. In Brazil, this condition is the second most prevalent chronic health condition, second only to systemic venous hypertension [2]. Recent estimates suggest a prevalence of 11.9%, and the overall number of individuals with low back pain considerably increases with the aging population [3]. The breadth of this condition’s socioeconomic impact and its effect on quality of life make it one of the greatest public health challenges worldwide [4-6].

Many efforts have been made to find effective treatments; however, back pain remains difficult to treat [7]. Conservative analgesic therapy usually comprises a variety of strategies, such as medication, acupuncture, kinesiotherapy, manual and behavioral therapy, multidisciplinary approaches and the use of physical methods such as transcutaneous electrical nerve stimulation (TENS) [8,9]. Despite being used for more than 20 years, TENS still presents controversial results, thus hampering its use as an effective treatment for chronic non-specific [10-13] low back pain. Many studies showing some evidence of TENS for pain relief show methodological flaws; in addition, the long-term benefits of this technique are not clear, as the effects occur especially during application [14,15].

New concepts like neuromatrix of Melzack [16] propose participation of brain mechanisms in pain chronicity, thus holding in check treatments that exclusively address peripheral structures. Moreover, studies focusing on neuroimaging showed structural and functional changes in the cerebral cortex of people with chronic musculoskeletal pain [17-19]. In the brain, sensorimotor control mechanisms therefore seem to contribute to the emergence and maintenance of the painful condition. Although some studies have shown that peripheral stimulation such as high frequency TENS can inhibit cortical excitability [20-22] this technique could be insufficient in modulating peripheral and central pain amplification, especially at encephalic levels, where “ill plastic adaptations” occur as a result of chronic low back pain [23-25].

Accordingly, treatments specifically addressing the cerebral cortex can complement the action of peripheral interventions and result in a more effective tool for the relief of chronic pain. The transcranial direct current stimulation (tDCS), despite not being a new modality, has presented recent advances in the treatment of chronic pain [26-30], establishing itself as a promising therapeutic tool. Like TENS, tDCS is a well-tolerated noninvasive low-cost technique with minimal side effects [31,32].

According to our literature review, there are only three studies involving the tDCS technique in chronic nonspecific low back pain: (1) a protocol study without prior results [33], (2) an exploratory study with only one application of tDCS [34] and (3) an exploratory randomized double-blind study [35]. Taken together, these studies showed no significant differences in pain relief between real and sham tDCS. Although the results may indicate that transcranial stimulation may not be enough to change the perception of pain, conclusions about the effectiveness of this technique in chronic low back pain should be cautious in view of the characteristics of the experiments. Transcranial stimulation associated with peripheral stimulation was investigated in only two studies. In patients with neurogenic pain in the arm, the combination of these techniques achieved greater reduction in pain than the isolated application of transcranial stimulation [36]. In a recent study, Siobhan and colleagues (2014) demonstrated that the combination of peripheral and transcranial stimulation was more effective in relieving the symptoms of chronic low back pain and mechanisms of cortical organization and sensitization than the interventions applied alone [37]. The promising results of these two cross-sectional clinical studies indicate the possibility of a synergistic action of the cortical and peripheral stimulation, and hence a more potent analgesic effect. However, there is a critical need for randomized clinical trials to generalize these findings and produce the best data available for clinical decision-making.

The identification of effective non-invasive and non-pharmacological treatments can trigger a substantial gain in pain relief and physical capacity, resulting in decreased morbidity and lower costs associated with low back pain. By simultaneously combining the two electrical stimulation techniques (cerebral and peripheral), the analgesic effect could be enhanced by the reorganization of cortical neuron activity and by the segmental inhibition of nociceptive stimulus. The aim of this experiment is to investigate whether cerebral and peripheral electrical stimulation combined are more effective in relieving pain than the isolated application of electrical stimulations in patients with chronic nonspecific low back pain.

## Objective

The primary objective of this study is to investigate the analgesic efficacy of transcranial (cerebral) direct current stimulation combined with transcutaneous (peripheral) electrical nerve stimulation in patients with chronic nonspecific low back pain compared with cerebral and peripheral electrical stimulations applied alone. The analgesic efficacy will be analyzed through the decrease of pain intensity four weeks after randomization.

The secondary objectives are:

To analyze the difference between cerebral, peripheral and combined stimulations in pain intensity in patients with chronic nonspecific low back pain assessed in Month 3 and in Month 6 after randomization.

To analyze the difference between cerebral, peripheral and combined stimulations in the sensory and affective aspects of pain in patients with chronic nonspecific low back pain assessed after four weeks, in Month 3 and in Month 6 after randomization.

To analyze the difference between cerebral, peripheral and combined stimulations in the functional capacity of patients with chronic nonspecific low back pain assessed after four weeks, in Month 3 and in Month 6 after randomization.

To analyze the difference between cerebral, peripheral and combined stimulations in the perception of the overall effect of patients with chronic nonspecific low back pain assessed after four weeks, in Month 3 and in Month 6 after randomization.

To analyze the patients’ satisfaction with the treatment.

## Hypothesis

Our hypothesis is that tDCS and TENS simultaneously combined are more effective in reducing the intensity and sensory aspect of pain, presenting less disability and having a better effect on global perception when compared to patients receiving the treatments alone.

## Methods/Design

**Figure 1:**
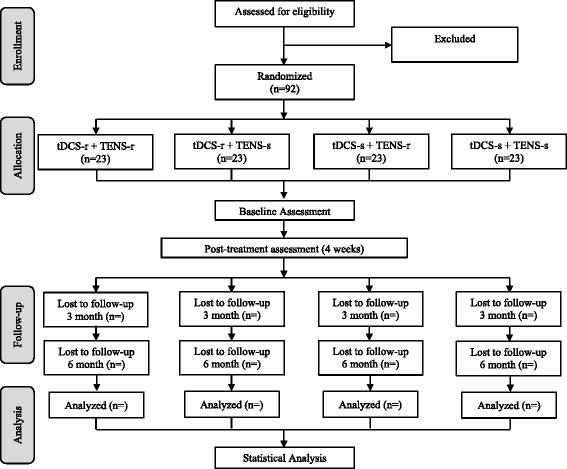
**Flow chart illustrating the process of the study.** tDCS and TENS indicate transcranial direct current stimulation and transcutaneous electrical nerve stimulation, respectively.

A controlled factorial (2 × 2) clinical trial will be conducted to evaluate the analgesic efficacy of cerebral and peripheral stimulation in chronic low back pain (Figure 1).

## Approval and registration

This project was approved by the Ethics Committee of the School of Medicine, University of São Paulo (USP) (protocol 308/13). The study will be conducted at the Clinical School of Physiotherapy, School of Medicine, USP, and at the Rehabilitation Center of the Irmandade da Santa Casa de Misericórdia de São Paulo, São Paulo, Brazil.

## Procedures

Patients with complaints of low back pain who seek treatment at the physiotherapy clinic of the School of Medicine, USP, and at the physiotherapy clinic of the Irmandade da Santa Casa de Misericórdia de São Paulo, will be initially evaluated by the medical staff of the institutions. After the diagnosis of chronic nonspecific low back pain and referral to the physiotherapy services, the patients will be thoroughly informed about the present study. The physiotherapist responsible for the evaluations will clarify the objectives of the study, possible treatments, eligibility criteria and potential risks resulting from the application of cerebral and peripheral stimulations. Patients who agree to the conditions and sign the consent form may participate. Participants who meet the eligibility criteria will be included in the study.

## Participants and eligibility

This study will involve 92 participants (both genders) who seek treatment at one of our rehabilitation centers through medical referral complaining of nonspecific low back pain for more than three months.

Participants who meet the following criteria will be eligible: (1) age between 18 and 65 years, (2) medical diagnosis of nonspecific low back pain present for at least three months, (3) spontaneous search for treatment and (4) signature of informed consent.

Participants will be excluded from the survey under the following conditions: (1) low back pain assessed <4 by Numeric Rating Scale (NRS) during one-week daily monitoring (patient registration); (2) previous spine surgery less than six months ago; (3) previous treatment with TENS less than six months ago; (4) previous tDCS treatment; (5) disk herniation with nerve compression with neurological compromise; (6) neurological, psychiatric and rheumatologic diseases; (7) use of pacemakers or other implanted devices; (8) pregnancy; (9) drug use and (10) abuse of medicines and alcoholic beverages.

The regular use of prescribed medications will not be an exclusion criterion, but the dose and type of medication will be recorded and documented. Patients with moderate psychiatric comorbidities such as anxiety and depression that are under medical supervision will not be excluded.

## Randomization and allocation concealment

Before starting treatment, a randomized generating program will place patients in one of the four groups: (1) real transcranial direct current stimulation (tDCS-r) + real transcutaneous electrical nerve stimulation (TENS-r), (2) real transcranial direct current stimulation (tDCS-r) + sham transcutaneous electrical nerve stimulation (TENS-s), (3) sham transcranial direct current stimulation (tDCS-s) + real transcutaneous electrical nerve stimulation (TENS-r) and (4) sham transcranial direct current stimulation (tDCS-s) + sham transcutaneous electrical nerve stimulation (TENS-s). The randomization and allocation concealment will be carried out by a collaborator not participating in the research, who should organize the patients’ records and their previously allocated treatments in individual opaque envelopes. The schedule of treatment will be revealed to the physiotherapist responsible for the treatments at the moment of electrical stimulation application. The “blinding” of evaluator and patients will be kept until the end of the research and data processing.

## Initial evaluation

After the fulfillment of eligibility and subsequent adherence and signature of the consent term, information on participants will be collected through a structured interview. Data will include personal data, clinical history and radiological findings (if any) and anthropometric characteristics. Subsequently, a clinical psycho-functional evaluation will be conducted according to the recommendations of the Initiative on Methods, Measurement, and Pain Assessment in Clinical Trials (IMMPACT) for clinical trials of effectiveness of treatment for chronic pain [38]. The main outcomes to be included in the clinical trials include six aspects: (1) pain, (2) physical functioning, (3) emotional functioning, (4) classification of improvement and patient’s satisfaction, (5) symptoms and adverse events and (6) patient’s approval.

## Primary variable

### Pain intensity

Pain intensity will be assessed on a numerical scale of 11 points (0–10), 0 accounting for no pain and 10 for the worst pain possible. The patient will be asked to describe the pain of the past seven days [39]. Pain intensity will also be assessed before and immediately after the application of protocols over the 12 sessions.

## Secondary variables

### Sensory and affective aspects of pain

The short version of the McGill Questionnaire (SF-MPQ) contains 15 descriptors of pain sensation (11 sensory and four affective), each descriptor rated on a 4-point scale where 0 = none, 15 = mild, 25 = moderate, and 35 = severe [40]. Three measurements of pain experience based on sensory and affective descriptors can be obtained: (1) PRI-T is the sum of all 15 descriptors with the total score ranging from 0 to 45, (2) PRI-S (sensory) is the sum of descriptors 1 through 11 with the total score ranging from 0 to 33 and (3) PRI-A (affective) consists of the sum of descriptors 12 to 15 with the total score ranging from 0 to 12. The SF-MPQ also includes the visual analog scale (VAS) and the present pain intensity (PPI) of the long version of McGill questionnaire.

### Physical functioning

The Roland Morris disability questionnaire assesses the overall functional capacity of the patient through the physical limitations resulting from low back pain. The questionnaire consists of 24 questions related to the normal activities of daily life, each affirmative answer corresponding to one point. The final score is determined by the sum of the scores. Values close to zero mean the best results, i.e. lower limitation, and values close to 24, the worst results, i.e. greater limitation. The highest number of responses is related to greater disability due to low back pain [39,41,42].

### Perception of global effect

The effect felt as a result of treatment will be assessed by the scale of global perceived effect that assesses the perceived level of the patient’s recovery from the treatment, comparing the initial symptoms with those of the last days. The resource is a numerical 11-point scale ranging from −5 to +5, with −5 = extremely ill, zero = no change, and +5 = completely recovered, with the highest score representing the fullest recovery [39].

## Other outcomes

### Emotional functioning - confusion factors

Depression and anxiety may be important confounding factors that influence the improvement of the patient’s pain. Thus, symptoms of depression and general anxiety will be assessed at the beginning and the end of treatment by the Brazilian version of the Beck Depression Inventory (BDI) [43] and Visual Analog Scale (VAS), respectively. The BDI is a tool of self-assessment of depression using a questionnaire with 21 items whose intensity varies from 0 to 3 (higher scores indicating more depressive symptoms). The VAS for general anxiety is assessed by a horizontal 100-mm-long line. The extreme left end points to no anxiety, and the extreme right end to the worst anxiety possible [44].

### Symptoms and adverse events

The record of symptoms and adverse events resulting from the use of tDCS and TENS is passively collected by the patient’s spontaneous reports. Active collection uses a questionnaire with records of the duration and intensity of adverse symptoms reported by the patient. Then the patients will be asked to rank the certainty of their claims according to a Likert scale (1 = no certainty and 5 = total certainty) [31].

### Patients’ approval

The approval of patients receiving treatment will be evaluated by the Medrisk questionnaire, which measures the satisfaction of patients undergoing physical therapy care. This questionnaire consists of 20 items, 10 of them related to the therapist-patient interaction, eight not related to it and two items considered overall items. The patient chooses his or her level of satisfaction with each item by selecting a Likert-like scale ranging from 1 (“strongly disagree”) to 5 (“strongly agree”) or by choosing option “not applicable,” where high scores represent high satisfaction [45].

### Evaluation of the blind condition

The evaluation of the blind condition of patients and the evaluator-therapist will be conducted at the end of four weeks of treatment. Both will be asked to guess which intervention was used for tDCS and TENS, whether real or sham. Then patient and evaluator will be required to rank the certainty of their claims according to a Likert-like scale (1 = no certainty, 5 = total certainty). To ensure that the evaluator is not induced to correctly guess the participants’ allocation, the latter will be instructed not to reveal which sensation was felt during the sessions.

### Treatments

The treatment will be carried out for four weeks, with three sessions per week, totaling 12 sessions of electrical stimulation. The sessions will take place individually in schedules of alternate days from Monday to Saturday. The program of schedules will allow a 20-minute interval between sessions so as to avoid possible contact between patients.

### Measurements of the participants’ expectations

After the enrollment and initial assessment (pre-treatment) and before randomization, the participants will be informed about the possible sensations arising from cerebral and peripheral stimulations. They will be informed that the unit of electrical stimulation may cause a slight tingling, itching or burning sensation the entire time or only at the beginning of application. The difference in sensations occurs according to the sensitivity of each individual. Participants will also be informed about the possibility of no perceptible sensation during the procedure. Regardless of the stimulus felt, all participants will be instructed not to reveal the sensation experienced.

### Transcranial Direct Current Stimulation (tDCS)

The transcranial stimulation is applied by a DC generator powered by a 9-volt battery (Activadose II, USA). The current will be applied using two electrodes (35 cm^2^; 5 × 7 cm) (Ibramed, Brazil) covered by a sponge damped with physiologic saline (salt solution 1%) and fixed to the head with elastic bands.

The assembly of the electrodes will be made according to the International 10–20 System of EEG [46] to better focus the primary motor cortex. The positively charged electrode (anode) will be placed at C3 or C4 (counterlateral to the side of pain complaint), and the negatively charged electrode (cathode) in the supraorbital ipsilateral region [29]. For patients experiencing pain in the central region of the lumbar spine, the anode will be placed on the side counterlateral to the dominant upper limb of the patient, as described in previous studies [47,48].

The groups that receive the real tDCS (tDCS-r) will be treated with electrical current intensity of 2 mA, density of 0.057 mA/cm^2^ and application time of 20 minutes [29]. The groups with sham tDCS (tDCS-s) will receive cerebral stimulation within the same parameters, but the application time will be only 30 seconds [35]. This feature of sham stimulation is usually used [47,48] because the tingling sensation on the skin below the electrode usually disappears after 30s. Thus, the placebo effect is more effectively monitored.

### **Transcutaneous Electrical Nerve Stimulation (TENS**)

The peripheral stimulation will be applied through a rectangular asymmetric two-phase electrical current apparatus (Neurodyn III Ibramed, Brazil). Both units of stimulation (real or sham) will be applied, using four self-adhesive electrodes (VALUTRODE 5 × 9 cm). The electrodes will be placed in a paralleled and bilateral position of the lumbar segment on the painful area.

The groups that will receive the real TENS (TENS-r) will be treated with current intensity (amplitude) according to their sensory threshold, characterized as an intense, yet comfortable, pulse frequency of 100 Hz, pulse duration of 200 μs and application time of 40 minutes [15]. During application, the patients will be asked about their perception of TENS intensity every five minutes and, when they get used to the sensation, the intensity will be increased again until the return of the sensory threshold.

Although some studies report no differences between high (100 Hz) and low (4 Hz) frequency for the treatment of chronic low back pain [13], prolonged use of opioid analgesics (activation of μ receptors) can induce tolerance to the drug and interfere with the effect of TENS, especially when low frequency is used [49]. Groups with sham nerve stimulation (TENS-s) will receive the same stimulation parameters, but the application time, like sham tDCS, will be only 30 seconds. The sham and the real TENS equipment will have the same appearance, but, after the initial 30 seconds, the current amplitude will gradually decrease to 15 seconds until it reaches zero, thereby stopping the issuance of the electric current. The equipment will remain inactive for the rest of the application, maintaining a light on during the whole process. The same occurs with the real stimulation machine.

### Sample size

The sample size was *a priori* estimated by the analysis of power [50] to detect a clinically important minimal difference (10% reduction from the baseline) in the outcome of pain intensity measured by the Numerical Rating Scale for Pain [51]. The effect size and the probability of error type 1 (α) and type 2 (β) were 0.33, 0.05 and 0.20, respectively. According to the data of sample calculation, there must be a total of 75 participants. However, considering 20% of sample loss, a sample size of 92 participants (23 per group) was calculated.

### Statistical analysis

All statistical procedures will be performed according to the principles of intention to treat. Linear mixed models will be used to identify intra- and inter-group differences at the end of treatment and in Month 3 and Month 6 of follow-up. All data will be analyzed using software SPSS v.20 for Windows. The level of significance is 0.05.

## Discussion

According to our review, this is the first randomized double-blind clinical trial to investigate the analgesic efficacy of transcranial direct current stimulation (tDCS) combined with transcutaneous electrical nerve stimulation (TENS) in patients with chronic nonspecific low back pain. The factorial design used in this study will allow three interventions to be evaluated simultaneously: cerebral stimulation, peripheral stimulation and combined stimulation. Furthermore, the use of sham tDCS and sham TENS represents a more robust experimental design to control the placebo effects. The results of this study will provide important information for clinical practice and conservative management of chronic low back pain. The possibility of synergistic and additive effect of these noninvasive, nonpharmacological, well-tolerated techniques presenting minimal adverse effects can turn them into a promising therapeutic tool for health professionals, improving the health status of the population and reducing the socioeconomic consequences of chronic low back pain.
